# Linguistic clusters in scoring semantic verbal fluency task in patients with pre‐dementia cognitive decline

**DOI:** 10.1002/alz70857_098530

**Published:** 2025-12-24

**Authors:** Nikita Cherkasov, Ekaterina Rodionova, Arina Zvereva, Svetlana Malyutina

**Affiliations:** ^1^ Mental Health Research Center, Moscow, Moskva, Russian Federation; ^2^ National Research University Higher School of Economics, Moscow, Moskva, Russian Federation

## Abstract

**Background:**

Previous works have shown that qualitative language characteristics of verbal fluency task may be useful in differentiating between different neurodegenerative states, such as: variants of frontotemporal dementia or primary progressive aphasia. There is only limited data on how qualitative parameters of verbal fluency are associated with pre‐dementia states, such as subjective cognitive decline (SCD) and mild cognitive impairment (MCI).

**Method:**

Semantic verbal fluency (SVF) task was given to 40 patients with SCD (median age 72 years, 39 females) and 51 MCI patients (median age 73 years, 43 females). Montreal cognitive assessment (MoCA) scores were obtained for all 91 patients. Phonemic and semantic linguistic clusters were derived from patient's SVF response using computerized approach. Qualitative language characheristics of linguistic clusters were calculated: number of switches between clusters, number of clusters, mean cluster size, first cluster size. Statistical analysis was performed and linear models with mixed effects were generated with MoCA and diagnosis as dependant variables and (i) routine SVF task score only (base model) vs. (ii) qualitative language characheristics and SVF task score (full model). Both models included age, sex and education level as covariates.

**Result:**

Patients with SCD and MCI had significant differences in SVF total score (14 [10; 16] vs. 10 [7; 12], *p* <0.001, ES=0.81 (0.45, 1.2)) as well as several cluster characteristics: phonemic number of switches (12 vs 9, *p* = 0.001), phonemic mean cluster size (2 vs 0, *p* = 0.006), semantic mean cluster size (4.8 vs 3.5, *p* = 0.007) and semantic first cluster size (5.0 vs 3.0, *p* = 0.003). ANOVA test demonstrated better prediction of MoCA score (AIC 812.76, BIC 847.88, χ^2^ = 0.035) and diagnosis (AIC 178.81, BIC 210.74, χ^2^ = 0.016) in the full model compared to the base model.

**Conclusion:**

Using novel qualitative language characheristics in verbal fluency task assessment might be a promising tool to improve diagnostic algorithm using computerized approach and better understand underlying language difficulties in pre‐dementia states.

